# 14. A great mimicking vasculitis

**DOI:** 10.1093/rap/rky033.006

**Published:** 2018-09-20

**Authors:** Simon Black, Charles Raine, Daniel Ivens, Richard Stratton

**Affiliations:** 1 Rheumatology, Royal Free Hospital, London, UNITED KINGDOM; 2 Sexual Health and HIV, Central and North West London NHS Foundation Trust, London, UNITED KINGDOM

**Introduction:** We report a case of a seemingly typical presentation of giant cell arteritis (GCA) with lack of response to steroid therapy which was, in fact, found to be due to underlying infection with syphilis causing a clinical syndrome with symptoms which mimicked GCA. Treatment of the infection led to complete resolution of the symptoms. Whilst syphilis is known to cause a wide range of multi-systemic symptoms, it is not usually considered in the different diagnosis in patients presenting with symptoms suggestive of GCA, as evidenced by the scarcity of previously documented cases.

**Case description:** A 64 year old male was referred to the infectious disease team by his GP with an eight-week history of drenching, intermittent night sweats, weight loss and low-grade raised inflammatory markers (CRP 37 mg/L, ESR 44 mm/hr). Screening for HIV and viral hepatitis was negative and there was no personal or contact history of TB nor any evidence of infectious or tropical disease. Autoantibody screen had been performed and ANA was found to be weakly positive at 1/100 but ENA, rheumatoid factor, anti-CCP and ANCA were all negative. CT of chest, abdomen and pelvis was normal however the patient subsequently reported a new-onset generalised headache. Inflammatory markers remained raised. On further detailed questioning, the patient reported some recent aching of the proximal upper limbs. He was hence referred for rheumatology opinion. Three weeks later, the patient presented to the emergency department with severe unilateral temporal headache, a sensation of burning of the scalp, hair loss and some visual blurring. Examination revealed a tender, thickened and hyperaesthetic temporal artery. Inflammatory markers remained elevated with ESR 32 mm/hr and CRP 20 mg/L. The impression was of PMR evolving into GCA and the patient was commenced on 60 mg daily of prednisolone. The patient was reviewed six days later and reported an improvement of his symptoms and a plan was made for weaning of the steroids. Temporal artery biopsy was performed and showed no evidence of temporal arteritis. One month later, the patient requested an urgent review due to a relapse of all of his symptoms. On this occasion, the patient was particularly concerned about worsening alopecia and more severe bilateral shoulder aching. Inflammatory markers remained at similar levels to those previous. His prednisolone was increased again and, it was thought his steroids had been weaned too rapidly. He was also referred to dermatology for an opinion regarding his hair loss. During the same period, the patient’s wife was referred to the genitourinary clinic for infective symptoms and the patient was subsequently requested for testing as a potential contact. It was noted in the sexual health clinic that the patient had additionally developed a mildly pruritic, faint, papular rash on both palms. Treponema pallidum particle assay (TPPA) was positive with a rapid plasma regain (RPR) titre of 1: 32. Secondary syphilis was confirmed and the patient was subsequently treated with intramuscular penicillin-G. Within two weeks, the patient reported rapid resolution of all his symptoms including the rash, hair loss, headache, scalp tenderness and shoulder pain. CRP was measured at 4 mg/L and ESR was 14 mm/hr. At the following clinic review, the patient had regained weight and steroids were tapered. The patient remained well at subsequent follow up (CRP <1, ESR 2) and continued to remain asymptomatic on no steroid therapy. One year on, he remains well with no recurrence of his symptoms.

**Discussion:** Syphilis has long been known as the great mimicker for its ability to present in a wide range of symptoms affecting multiple systems. Secondary syphilis represents haematogenous dissemination from the initial site of infection (as sometimes represented by a primary chancre) and most commonly presents with skin rashes, alopecia and lymphadenopathy. Late or tertiary syphilis often occurs two or more years after acquisition and causes gumma formation, aortitis and neurological involvement such as focal arteritis causing infarction and meningeal inflammation. This case represents a rare case of secondary syphilis presenting initially with symptoms highly suggestive of classic giant cell arteritis. Only two previous such cases have been reported in the literature. Such was the clear constellation of symptoms, the patient was treated with high dose oral steroids as is common practice but tellingly failed to respond accordingly and, indeed, subsequently experienced a worsening of symptoms. The only initial clue regarding the atypical nature of this case, prior to the later development of the palmar rash, was the prominent alopecia which is described in secondary syphilis. Tertiary neurosyphilis with meningeal involvement is rare but would present with additional neurological symptoms such a seizure or cranial nerve palsy. The mechanism behind secondary infection causing headache and a mimicking of giant cell arteritis is unclear and a negative temporal artery biopsy would not include or exclude the condition regardless. Whilst an extremely rare manifestation, this case is interesting in being a reminder that other systemic infections can often present similarly to inflammatory conditions seen within rheumatology. Secondary syphilis should be considered within the differential diagnosis of patients presenting with symptoms suggestive of GCA, especially in atypical cases or when there is a lack of response to steroid therapy.

**Key Learning Points:** Include multi-systemic infection within the differential for patients presenting with symptoms consistent with inflammatory of or connective tissue disease. Exclude other causes when giant cell arteritis remains unresponsive to conventional treatment.

**Disclosure: S. Black:** None. **C. Raine:** None. **D. Ivens:** None. **R. Stratton:** None.

